# Analgosedation for diagnostic and interventional procedures: a countrywide survey of pediatric centers in Germany

**DOI:** 10.1186/s13052-020-0783-y

**Published:** 2020-02-03

**Authors:** Harald Sauer, Marie Lobenhofer, Hashim Abdul-Khaliq

**Affiliations:** 1 Department of Pediatric Cardiology, University Hospital of Saarland, Kirrberger Strasse, Building 9, 66421 Homburg (Saar), Germany; 2 Department of Gynecology, St. Theresa Hospital Nuremberg, Nuremberg, Germany

**Keywords:** Sedation analgesia, Diagnostic procedures, Interventional procedures, Pediatric centers, Survey

## Abstract

**Background:**

As more and more diagnostic and interventional options are becoming available for use in pediatric patients, techniques of procedural sedation analgesia (PSA) are being administered in considerably growing numbers as well.

**Aims:**

The objective of this research effort was to conduct the first countrywide survey on the status quo of sedation analgesia as delivered to children and adolescents in Germany.

**Methods:**

We dispatched letters to all pediatric hospital settings in Germany (*n* = 305), including a questionnaire that had been developed with existing guidelines taken into account. Its items were designed to elucidate the current practice of PSA throughout these pediatric centers regarding (a) organizational structures and (b) standards of medication and staffing.

**Results:**

A total of 138 centers returned the questionnaire, hence the response rate was 45.2%. Numerous centers had implemented adequate structures and staffing standards. Deficits were nevertheless identified, most notably in terms of on-location equipment and staff provided to deliver sedations. Essential items of equipment were not provided in up to 26.8% of centers. Adequate staffing was not provided in up to 44.2% of centers, depending on the diagnostic or interventional procedures for which the PSA was delivered. The most widely used sedative agents were midazolam, ketamine/esketamine, and propofol.

**Conclusions:**

Adequate care structures for the management of procedural sedation analgesia have been implemented by many pediatric centers in Germany. On the downside, these findings also reveal deficits that will take efforts to be eliminated.

## Introduction

The past few years have seen increasing attention devoted to the topic of procedural sedation analgesia (PSA) in pediatric patients [1]. Even premature babies and newborns are today eligible for a wide range of diagnostic and/or interventional procedures such as computed tomography, magnetic resonance imaging, a wide range of endoscopic interventions, organ biopsies, or catheter examinations. As more and more of these procedures are being performed on children and adolescents in clinical practice, the administration of sedation and analgesia in this patient group has grown accordingly [1–4].

While this growing requirement has engendered many publications, the guidelines and recommendations developed on their basis do exhibit discrepancies in specific areas [5, 6]. Both the experience at our own center and discussions with colleagues from other parts of Germany have suggested to us that there is an increasing need for pediatricians to carry out techniques of PSA by themselves, given an inability of anesthesiologists to keep up with this growing demand.

Therefore we designed the first countrywide survey on the status quo of PSA administered to children and adolescents. This was to be accomplished by developing a questionnaire and sending it to all pediatric hospital settings, with the goal of evaluating the degree to which the relevant current national and international recommendations have been implemented throughout Germany.

## Methods

### Questionnaire and handling of the survey

The questionnaire was developed on the basis of existing national and international guidelines and publications, also taking into consideration our own in-house standard [6]. Its purpose was to illustrate management practices before, during and after PSA. This questionnaire was enclosed with letters that we sent to all pediatric centers listed on the DGKJ (German Society of Pediatrics and Adolescent Medicine) website,^1^ asking these to fill it out either on paper or online. Contact was attempted for three times. Whenever a center had distinct units, we contacted each unit separately and later consolidated, for analysis, any multiple unit-level responses to center-level responses. Every center was assigned one specific random code number to prevent double responses.

### Major topics covered by the questionnaire

Part 1 of the questionnaire dealt with organizational structures. Major items concerned the formal level of care provided by each pediatric center, the settings used for PSA, the number of sedations conducted per year, the existence of sedation teams, of regular training courses for sedation, of workflows defined in writing, as well as the degree of equipment available in the sedation environments. Part 2 dealt with the medication and staffing standards implemented in the centers, focusing on the practical delivery of PSA in general, on specific examination scenarios in particular, as well as on training and safety requirements. The survey did neither contain any questions about the local standard for fasting nor the implementation of national or international fasting guidelines.

### Diagnostic and interventional procedures for sedation

**Fig. 1:**
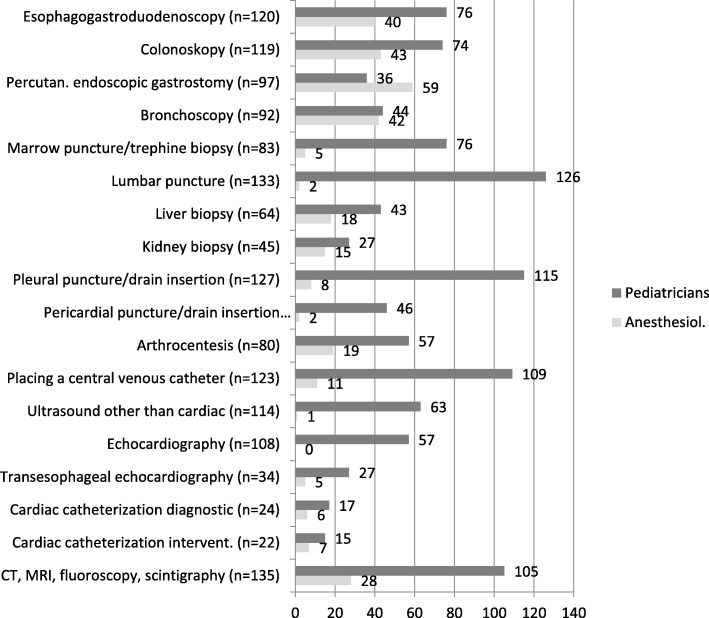
Graph illustrating how many of the responding centers (*n* = 138) perform each of the listed 18 diagnostic and/or interventional procedures and how many of the sedations accompanying each procedure are carried out by either pediatricians or anesthesiologists. Due to incomplete replies, any two figures for a pair of bars may not add up to the sum given on the left

**Fig. 2:**
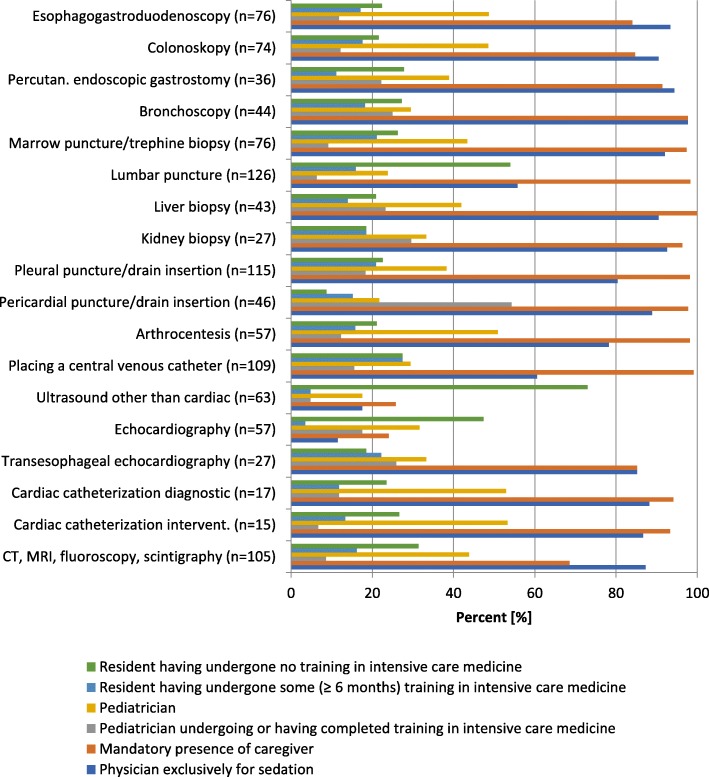
Graph considering only the subset of responding centers (*n* = × × ×) stating that pediatricians (rather than anesthesiologists) were in charge of a specific sedation scenario. Horizontal bars illustrate the standby personnel provided by the centers for each of the 18 diagnostic and/or interventional procedures and the requirements they impose on the qualification of the pediatricians in charge of the sedation

Eighteen scenarios of PSA (also see Figs. 1 and 2) were considered: esophagogastro(duodeno)scopy, colonoscopy, percutaneous endoscopic gastrostomy, bronchoscopy, bone marrow aspiration and/or trephine biopsy, lumbar puncture, liver biopsy, kidney biopsy, pleural puncture/pleural drain insertion, pericardial puncture/pericardial drain insertion, arthrocentesis, placement of a central venous catheter, ultrasound examinations other than cardiac, echocardiography, transesophageal echocardiography, diagnostic cardiac catheterization, interventional cardiac catheterization, and imaging techniques other than ultrasound (computed tomography, magnetic resonance, fluoroscopy, scintigraphy).

### Staffing and professional requirements for sedation delivery

We asked which sedation scenarios were handled by either an anesthesiologist or a pediatrician. In the latter case, we asked about the professional qualification that the center demanded as a minimum requirement, distinguishing between four levels: resident with immediate access to a regular pediatrician or a supervisory attending physician; resident having undergone ≥6 months of training in intensive care medicine; regular pediatrician; or regular pediatrician undergoing or having completed training in intensive care medicine. We also asked for each sedation scenario whether a nurse was mandatorily present and whether the physician in charge of the sedation was different from the physician in charge of the procedure (both being strictly yes-or-no questions).

### Adaptation of professional requirements to risk factors

Questions were also included to find out about organizational structures that involve a risk-adapted approach, meaning that the presence of patient-related risk factors would entail a requirement for sedation to be performed by higher-level physicians. Three risk factors were considered for this purpose: age (five age groups), severity of underlying disease as per the ASA classification [7, 8], and abnormal respiratory findings/potential airway complications (four categories). Two more items at the end of the questionnaire were used to find out about settings for, and monitoring during, the wake-up phase.

### “Large” versus “small” pediatric centers

Levels of care are used in a nomenclature describing the services offered by hospitals in Germany. We adopted this classification and accordingly distinguished between centers of primary care, general care, focus care, maximum care, and university departments for this survey. To find out whether the organizational structures for sedation correlated with the level of care offered by a center, we pooled centers of maximum care and university departments into “large” centers and compared these to “small” ones pooled from centers of primary, general, and focus care. Monte Carlo sampling was used to calculate approximate values and to draw conclusions at the *p* < 0.05 significance level. All data analysis was performed using IBM SPSS (version 22) statistics software.

## Results

### Response to the survey, basic organizational data, case-history considerations

We contacted all 305 pediatric centers listed on the DGKJ website. A total of 161 questionnaires were returned. Multiple responses from 18 centers that had more than one unit were consolidated to center-level responses. This resulted in 138 responding centers, meaning that the overall response rate was 45.2%. Table 1 summarizes basic and organizational data about the responding centers. In addition, these had also been asked whether they gave consideration to specific factors in their patients’ histories relevant to minimizing the risk of sedation. Given the unequivocal nature of these questions, any omitted replies were analyzed as negative replies. No consideration was given to the first group of these factors (allergies or intolerances to sedatives or anesthesia; sedation-related adverse events experienced in the past by the patients or their family members) in 15.9% and to the second group (infections, and notably airway infections, within 2 weeks before the procedure) in 18.1% of centers.

**Table 1:** Basic data and organizational structures for sedation analgesia as carried out in pediatric centers across Germany (based on 134 out of 138 responding centers; in the remaining four centers, no sedation analgesia was performed by pediatricians)

Center-specific levels of care	
Primary care	3.6%
General care	21.7%
Focus care	30.4%
Maximum care	25.4%
University departments	18.1%
Settings for sedation analgesia	
Pediatric ward	87%
Intensive care unit	76.8%
Operating room	13%
External/other settings	79%
Frequency of sedation analgesia	
> 150 per year	45.8%
100–150 per year	13.7%
50–100 per year	24.4%
< 50 per year	16%
Organizational structures	
Sedation teams present	40.5%
Regular sedation training courses	32.3%
Documentation protocol for sedation	80.6%
Post-sedation clinical examination	78.4%
Settings for the wake-up phase	
(General) pediatric ward	56.5%
Intensive care unit	11.6%
Wake-up room	11.6%
No commitment	20.3%
Parameters monitored in the wake-up phase	
Pulse oxymetry (mandatorily)	99.3%
ECG (mandatorily)	55%
RR (mandatorily)	89.9%
RR every 5 min: 21.8%; 10 min: 41.1%; > 10 min: 20.2%; beginning and end only: 16.9%	

### Equipment for pediatric sedation, medications, techniques, delivery by disciplines

Table 2 summarizes the on-location equipment provided by the responding centers with a specific focus on essential equipment. Again, any omitted replies to unequivocal questions in this regard were considered negative replies. Around 5% of the centers did not (contrary to existing recommendations) provide for on-location oxygen supply and pulse oxymetry. All other essential equipment was absent in many more environments, ranging from 15.9 to 26.8% of centers. Similar replies were obtained for the MRI sedation environments not included in Table 2, although more equipment other than for ECG and RR was present in this situation. Table 3 summarizes how many of the centers used which medications for sedation (and analgesia). Figure 1 illustrates which of the 18 diagnostic or interventional procedures were performed in the 138 pediatric care centers (not every procedure was offered by all) and how frequently the sedation was delivered either by pediatricians or anesthesiologists. Note that 17 of these procedures (with the exception of percutaneous endoscopic gastrostomy) were predominantly conducted with the sedation (analgesia) delivered by pediatricians.

**Table 2:** Equipment of the sedation environments used by the responding pediatric centers (*n* = 134)

On-location items	Always present	Available if required	Not available	Essential^a^
Oxygen	94.2%	2.2%	0%	yes
Compressed air	80.4%	10.1%	3.6%	yes
Suction	84.1%	10.1%	0.7%	yes
Electrocardiography	76.1%	18.1%	0.7%	yes
Blood pressure (RR)	73.2%	20.4%	0,7%	yes
Pulse oxymetry	94.2%	2.2%	0%	yes
Capnometry	5.8%	43.5%	31.2%	no
Emergency^b^	84.1%	11.6%	0%	yes
Defibrillator	26.1%	59.4%	7.2%	no
Ventilator	17.4%	65.9%	10.1%	no

Note that MRI environments are not included in this overview

^a^Essential equipment (modified in accordance with 7, 8)

^b^Emergency care equipment, including intubation instruments

**Table 3:** Popularity of agents used for sedation analgesia in pediatric centers across Germany (*n* = 134)

Agents	Centers
Midazolam	97.8%
Ketamine^b^	86.2%
Propofol	84.8%
Fentanyl	42.0%
Chloral hydrate	32.6%
Piritramide	31.1%
Morphine	29.7%
Diazepam	21.7%
Phenobarbital	21.0%
Livopan®	18.8%
Remifentanil	16.7%
Etomidate	12.3%
4-hydroxybutanoic acid^a^	10.9%
Pethidine	6.5%
Alfentanil	4.3%
Promethazine	4.3%
Other benzodiazepines	4.3%
Sufentanil	3.6%
Thiopental	2.9%
Other opioids	2.9%
Haloperidol	1.4%
Melatonin	1.4%
Dexmedetomidine	0.7%
Chlorprothixene	0.7%
Other:	8.7%

^a^4-hydroxybutanoic acid/γ-hydroxybutyrate

^b^Ketamine/esketamine

### Staffing for sedation, qualification levels, dual-role acceptance, adaptation to risk

Figure 2 illustrates for these 18 diagnostic and interventional procedures the minimum levels of professional qualification required for pediatricians delivering the sedation. The picture obtained varies. Most sedations were delivered by regular pediatricians rather than residents, the only exception being sedations for those procedures that are usually learned during residency (e.g. lumbar puncture and non-invasive techniques). A nurse was usually present to assist in around 95% of centers, exceptions here being esophagogastroduodenoscopy, colonoscopy, transesophageal echocardiography, non-sonographic imaging, and non-invasive sonography. Up to 44.2% of centers accepted “dual roles” with the same pediatrician delivering the sedation and performing the diagnostic or interventional procedure (again, this was strictly a yes-or-no question so that any omitted replies were considered negative replies). Dual-role acceptance by centers was seen for all 18 procedures. Regarding the question of centers linking case-specific risk factors to a requirement that higher-level (more experienced) physicians should then be in charge of the sedations, it emerged that around half of the centers did have such risk-adapted rules in place for each of the three specified risk factors.

### Differences associated with levels of care (“large” versus “small” pediatric centers)

No significant differences between large and small centers were seen regarding the provision of standby sedation teams (*p* = 0.216) or regular sedation training courses (*p* = 0.341). Large centers were found to use intensive care units significantly more often for PSA than small centers (*p* < 0.001). No differences were seen for any of the other settings (pediatric ward, operating room, external/other). In addition, we correlated levels of care with staffing standards for the seven most common sedation scenarios in pediatric centers (esophagogastroduodenoscopy, colonoscopy, lumbar puncture, bone-marrow aspiration/trephine biopsy, pleural puncture/pleural drain insertion, placement of central venous catheter, imaging techniques other than ultrasound). None of these scenarios revealed any significant between-center differences regarding the presence of a nurse. Dual-role acceptance, however, was found to be significantly more prevalent in small centers (15.2%) than in large ones (0%) for sedations in conjunction with colonoscopy (*p* = 0.04). The reverse was true of bone-marrow aspiration/trephine biopsies, where the same physician was found to be also in charge of the sedations in 13.6% of large versus 0% of small centers (*p* = 0.039).

## Discussion

This countrywide survey is the first study to yield an overview of how sedations for diagnostic and/or interventional procedures are provided to children and adolescents in Germany. Its reasonable response rate of 45.2% illustrates the importance attributed to this topic. Around 60% of the responding centers delivered at least two sedations per week. While this survey captured merely a snapshot and not a trend, the literature does provide evidence for PSA becoming increasingly common [1–4].

We agree with recommendations and demands raised in numerous publications that a standardized approach is mandatory [7–9]. Given the additional provision of adequate on-location equipment and well-qualified staff, patient safety can be ensured and the quality of sedation optimized. Our findings show that specialized sedation teams are established in over 40% of pediatric centers. Thus we have come a long way, but there continues to be room for improvement. Regular training is demonstrably helpful in implementing standardized processes [6]. It is therefore all the more surprising that only around one-third of the responding centers organized such courses recurrently. A need for regular trainings and organizational improvements can also be postulated from other findings: 15.9% of pediatric centers did not ask their patients if they were allergic or intolerant to substances used for PSA, around 20% did not consider airway infections during the pre-sedative period, one-fifth did not use a standardized documentation protocol, and one-fourth did not perform follow-up examinations.

In its latest update on how to deliver safe sedation to children, the AAP (American Academy of Pediatrics) has pointed out that adverse events and adequate organization are inversely related. Hence the AAP expressly recommends that each patient’s health, as well as specific risk factors that might be relevant to an uneventful course of PSA should be evaluted [10]. German law requires a record of the PSA process and of any clinical steps, to be taken in the form of an adequate documentation protocol. Several papers have been devoted to the need for comprehensive documentation [8, 10–12]. Our survey reveals deficits even regarding the essential items of equipment in pediatric sedation environments, and what options are available are not utilized in a consistent fashion. It has repeatedly been highlighted that both adequately equipped sedation settings and appropriate monitoring are mandatory [5, 7, 8, 10, 11, 13]. There is a need to further raise awareness of this problem and to eliminate deficits as required.

A wide range of agents were used for sedation analgesia by the responding centers. We would not recommend specific agents for specific procedures. While this restraint is consistent with the stance taken by the AAP [7], it is in contrast to the DGAI’s (German Society of Anesthesiology and Intensive Care Medicine) and BDA’s (Association of German Anesthesiologists) approach [11]. ASA (American Society of Anesthesiologists) issues general recommendations [14] while NICE (National Institute for Health and Care Excellence) does recommend specific agents [8]. Propofol is a good example to illustrate controversial discussions of the past few years about sedation delivery by non-anesthesiologists [15–17]. A consensus paper issued by 21 European national societies of anaesthesia is, in fact, entitled “Non-anaesthesiologists should not be allowed to administer propofol for procedural sedation” [18]. The other side of the story is illustrated by two exemplary reports covering approximately 50000 [19] or 25000 [20] patients in North America, both concluding that severe complications from propofol are quite rare. This was substantially confirmed by Chiaretti et al. in the largest European study in more than 36,500 patients with propofol administration for PSA in children [21].

That said, certain conditions must be met for the administration of propofol. We agree with the above publications in that we, too, discern mandatory requirements for a standardized approach, for adequate on-location equipment, and for anyone delivering sedations having an appropriate level of training—including but not limited to airway and emergency management and a solid understanding of the medications used. Our results show that many centers are aware of inadequate training being a problem and have implemented risk-adapted rules for sedation. Yet the responses also show that there is considerable room for improvement. What remains generally unanswered is the question just how much training should be required from an anesthesiologist or pediatrician to be considered “experienced enough” for safe delivery of a sedation, regardless of its depth [14]. While we have repeatedly reported on the good experience with our own in-house standard [5, 6, 22, 23], any wholesale extrapolation of this experience and its analysis would be misguided, given that other pediatric centers may have training programs that differ from ours in content and emphasis. Further corroboration of the above points comes from impressive evidence by Hoffmann et al. [24] for an association between rigorous adherence to predefined standards and complication rates.

Besides any requirements for adequate training of the physicians and nurses carrying out and/or assisting in sedations, there is also the question of how the on-location staff should be structured. The current guidelines repeatedly point out that performing a diagnostic or interventional procedure and delivering the accompanying PSA are incompatible activities in the hands of the same person [7, 8, 10, 11]. The DGAI and BDA require that, in severely diseased (ASA status III-IV) children and during all deep sedations, a second physician not identical to the examiner must be available who is trained in anesthesiology or intensive care medicine and whose sole task is to continuously monitor the vital parameters [11]. The AAP recommends the presence of both an examiner and a helper even in scenarios of moderate sedation, with the helper, once the sedative agents have been administered, monitoring the vital parameters and assisting with tasks of short duration; in scenarios of deep sedation, this specially trained person should not have any tasks other than activities related to the additional delivery of sedative agents or monitoring the sedation [7, 10].

We were surprised against this background that, regardless of which diagnostic or interventional procedure we considered, there were always some pediatric centers that organized this procedure without an assistant for the sedation part. In some cases, organizational deficits were indeed found in the two-digit percentage range. In addition, numerous centers affirmed that they accepted “dual roles” (meaning that the physician doing the procedure is also in charge of delivering the PSA) for a considerable number of the 18 diagnostic or interventional procedures listed in the questionnaire. Thus we could identify a considerable need to eliminate deficits in both of these areas (no assistant and dual-role acceptance) if the recommendations issued by the aforementioned professional associations are to be met. We believe that any deep sedation must involve two physicians (one being exclusively in charge of the sedation and the other one conducting the diagnostic or interventional procedure) and that any moderate level of sedation should not be delivered without an assistant. Exceptions are only possible under certain conditions, e. g. if a second non-physician has received adequate training in performing and monitoring PSA and the safety of the patients is not adversely affected.

Regarding limitations of our survey, certainly the questionnaire was rather long. For this reason, the important topics of local standards for fasting or the implementation of fasting guidelines were not addressed, nor were the rates of adverse events or complications. Even though many items just required boxes to be checked, any respondents would take around 20 min to complete it. This may also explain why items were sporadically omitted. Perhaps also due to this long questionnaire, the return rate of around 50% surely could have been higher. Another limitation is that, despite our reassurance that the replies would be processed in a pseudonymized format, a certain risk of dishonesty on the part of some respondents cannot be dismissed out of hand but, at the least, seems unlikely when considering that the survey disclosed some rather serious deficits.

## Conclusions

The growing demand for pediatric PSA to accompany diagnostic and interventional procedures poses a major challenge to the medical personnel in charge of treatment. Establishing adequate structures and staffing standards is indispensable for the safety of the children and adolescents entrusted to us. While our countrywide survey demonstrates that many pediatric centers have already made appropriate provisions, its findings also reveal shortcomings whose elimination will require suitable efforts. Adequate in-house standards and equipment of the sedation settings, as well as the provision of enough appropriately qualified staff and of regular training courses are the cornerstones required for a well-functioning system of PSA.

On the national level, we believe that there is an urgent need for the three major German associations (DGKJ, DGAI, BDA) to come up with a joint set of guidelines. The next requirement will be to maintain and actively support organizational structures, as well as to address and eliminate deficits, in accordance with these joint guidelines. On the international level, the extent to which the results of our countrywide survey can be extrapolated to other countries remains speculative, given that we are unaware of any other nearly as comprehensive surveys on pediatric PSA in the literature. Nevertheless, our results can point into the right direction to use the positive and negative results as a basis of a specific evaluation and correction, as necessary. Another way of viewing our results is that they highlight, based on evidence, the challenges of pediatric sedation analgesia, all boiling down to this quote by Gozal and Mason [15]: *“The challenge facing sedation care providers moving forward in the 21st century will be to determine how to apply the local, regional and national guidelines to the individual sedation practices. A greater challenge, perhaps impossible, will be to determine whether the sedation community can come together worldwide to develop standards, guidelines and recommendations for safe sedation practice.”*

## Data Availability

Data and materials are available via the department of Pediatric Cardiology of the University Hospital of the Saarland. Furthermore, the data and materials pertain to a doctoral thesis, which is available via the deanship of the Faculty of Medicine of the University of Saarland.

## Footnotes

1 https://www.dgkj.de/veroeffentlichungen/kinderkliniken/
